# Impact of Adjunctive Laser Irradiation on the Bacterial Load of Dental Root Canals: A Randomized Controlled Clinical Trial

**DOI:** 10.3390/antibiotics10121557

**Published:** 2021-12-20

**Authors:** Johannes-Simon Wenzler, Wolfgang Falk, Roland Frankenberger, Andreas Braun

**Affiliations:** 1 Department of Operative Dentistry, Periodontology and Preventive Dentistry, Rheinisch-Westfälische Technische Hochschule University Hospital, Pauwelsstrasse 30, 52074 Aachen, Germany; anbraun@ukaachen.de; 2 Department of Operative Dentistry, Endodontics and Pediatric Dentistry, Campus Marburg, University Medical Center Giessen and Marburg, Georg-Voigt-Str. 3, 35039 Marburg, Germany; frankbg@med.uni-marburg.de; 3 Center for Dental Microbiology, Bergstr. 26, 24103 Kiel, Germany; wolfgang.falk@web.de

**Keywords:** semiconductor laser, endodontics, bacterial reduction, sodium hypochlorite, disinfection

## Abstract

Successful root canal treatment depends on the adequate elimination of pathogenic bacteria. This study evaluated the effectiveness of a novel 445-nm semiconductor laser in reducing bacteria after chemomechanical root canal treatment. Microbiological specimens from 57 patients were collected after emergency endodontic treatment, in the following sequence: 1, removal of the temporary filling material; 2, chemomechanical treatment; 3, rinsing with sodium hypochlorite (3%) along with one of three adjuvant protocols (*n* = 19 in each group). The adjuvant procedures were: (a) sodium hypochlorite rinsing alone (3%); (b) laser irradiation; (c) combined sodium hypochlorite rinsing and laser irradiation. The diode laser was set to 0.59 W in continuous-wave mode (CW) for 4 × 10 s. After the flooding of the root canal with saline, specimens were collected using paper points and analyzed microbiologically. Statistically significant reductions in the bacterial load were observed in all three groups (*p* < 0.05): 80.5% with sodium hypochlorite rinsing alone and 58.2% with laser therapy. Both results were lower than with the combination of sodium hypochlorite rinsing and 445-nm laser irradiation, at 92.7% (*p* < 0.05). Additional disinfection of the root canal can thus be achieved with 445-nm laser irradiation after conventional chemical disinfection with sodium hypochlorite solution.

## 1. Introduction

Almost all conditions that require endodontic treatment are caused by, or later exacerbated by, microbial infections [1]. Between 10^2^ and 10^7^ bacteria can be detected in an infected root canal system [2]. The migration of these bacteria into all parts of the root canal system and also into the surrounding dentin makes it almost impossible to eliminate them completely. Insufficient reduction of bacteria in the root canal system is, therefore, one of the main reasons for the failure of endodontic treatments. The mechanical removal of infected hard and soft tissue during the shaping of the root canal for subsequent obturation with a suitable filling material leads to a considerable reduction in microorganisms. Still, disinfection of the root canal system is extremely important for systematic root canal treatment. In particular, the cleaning of hard-to-reach parts of the canal sections, mechanical instrumentation of inaccessible side canals, and thorough disinfection of the remaining surrounding dentin are decisive factors that determine the lasting success of endodontic treatment [3]. Rinsing solutions such as sodium hypochlorite (NaOCl), chlorhexidine digluconate (CHX), and ethylenediaminetetraacetic acid (EDTA) are generally used for this purpose. In combination with the mechanical instrumentation of the canal lumen, carefully performed chemomechanical root canal preparation can eliminate over 95% of the microorganisms in the root canal system [4,5].

In the literature, conventional rinsing with sodium hypochlorite has been reported to be effective in killing bacteria up to dentin depths of 160 µm [6,7]. However, this may not be sufficiently effective in relation to highly resistant bacteria such as *Enterococcus faecalis*, which reach root dentin penetration depths of more than 1000 µm [6,8]. Several methods have been developed to improve the penetration depth of disinfection measures to eliminate bacteria from deep residual root dentin, such as ultrasound devices, hydrodynamic rinsing methods, and laser-based procedures [9,10,11]. In addition to the positive effects of greater penetration depths reported in the literature, disadvantages have also been described with some procedures. Particularly with mechanical procedures, a risk of apical extrusion and resulting damage to apical tissues has been reported [12].

Nonmechanical approaches, such as thermally acting laser systems, have therefore also been investigated [13]. These can supplement conventional disinfection methods as adjunctive antimicrobial procedures. It should be noted, however, that laser effects depend on the laser wavelength and power settings used. To date, only a few studies have been conducted to assess the effectiveness of laser irradiation for endodontic disinfection purposes. To the best of the authors’ knowledge, there have been no studies on the blue laser wavelength of 445 nm that has recently been introduced in the field of dentistry. The aim of the present in vivo study was, therefore, to investigate the disinfectant effect of a 445-nm diode laser as an adjuvant therapy option, testing the hypothesis that adjuvant laser irradiation additionally reduces the residual bacterial count after conventional root canal disinfection.

## 2. Materials and Methods

As part of the regular endodontic treatment regimen, microbiological specimens were collected from 57 patients and microbiologically evaluated. All of the patients had provided informed consent to participation. The study was conducted in full accordance with established ethical principles (World Medical Association Declaration of Helsinki, version VI, 2002) and was approved by the local ethics committee (reference number 016/1749). Only teeth with suspected irreversible pulpal disease were selected according to the diagnostic criteria based on clinical symptoms and radiographic findings. The teeth exhibited an increased stimulus response or spontaneous pain, as well as prolonged pain episodes up to continuous pain. Radiographically, no or minimal radiographic changes were evident. Prolonged pain on cold contact was present, and increased bleeding from the root canal was noted after trepanation. Teeth with pulp necrosis were excluded. The routine treatment of the teeth involves the following steps: trepanation, mechanical root canal treatment (rinsed conventionally using sodium hypochlorite), an intermediate temporary filling, and finally definitive filling of the root canal after 1 week. In the present study design, all of these steps were performed in all of the study arms.

### 2.1. Patient Selection

The study included patients attending the Department of Operative Dentistry and Endodontology at the University of Marburg who had endodontically induced pain and had been diagnosed with irreversible pulpitis. Inclusion criteria were a minimum age of 18 years, teeth with irreversible pulp disease but with an endodontically preservable root structure, and a written declaration of consent. Exclusion criteria were acute pain after removal of the pulp tissue, antibiotic treatment within the previous 6 months, probing depths indicating a periodontal-endodontic lesion, previous endodontic treatment of the affected tooth, and pregnancy. After the application of these inclusion and exclusion criteria, 57 patients were included in the study.

Following endodontic pain treatment, including rubber dam isolation, trepanation of the pain-triggering tooth, and conventional rinsing with sodium hypochlorite (3%, 5 mL, 1 min), temporary calcium hydroxide paste was filled into the canal and temporarily sealed. The patients were then randomly assigned to the different groups in the clinical trial.

### 2.2. Treatment Procedure

One week after emergency endodontic treatment, the tooth was isolated with a rubber dam, and its surface was disinfected with hydrogen peroxide solution (30%; Carl Roth GmbH, Karlsruhe, Germany). The rubber dam was then disinfected with Lugol’s iodine solution (5%) and inactivated with sodium thiosulfate (5%; Dr. Franz Köhler Chemie GmbH, Bensheim, Germany) so that any residue on it would not influence the bacteriological sampling.

**Figure 1:**
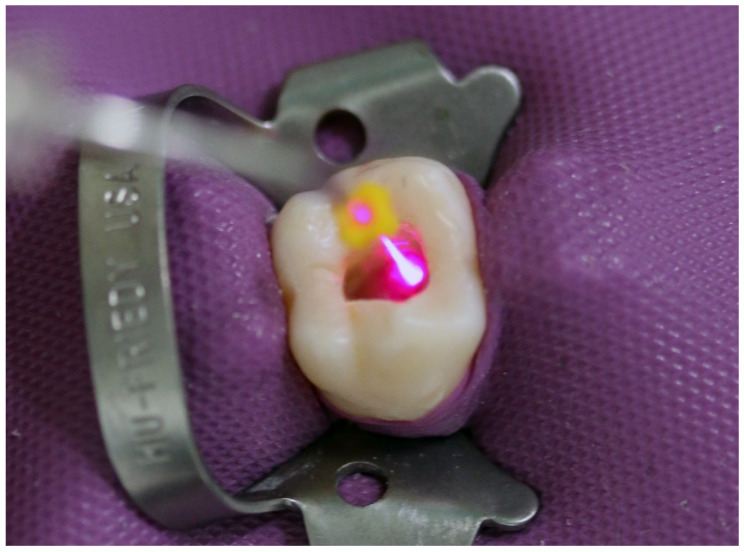
Representative picture of adjunctive laser disinfection during root canal treatment.

In accordance with the study protocol, the following treatment sequence was performed, and microbiological samples were taken at three time points using sterile paper points (ISO 30; VDW Antaeos GmbH, Munich, Germany), which were left in the root canals flooded with saline solution for 1 min each:

1. Removal of the temporary filling material.
2. Chemomechanical preparation was performed manually to size 25.02 using hand files in a pulling movement only (Hedström, VDW GmbH, Munich, Germany) and mechanically to size 30.09 (ProTaper Gold F1-F3 employing SiroNiti Apex endodontic handpiece; Dentsply Sirona GmbH, Bensheim, Germany) along the glide path with each insertion deeper than the previous one until the working length was reached. The root canal system was irrigated with sodium hypochlorite (3%; total 5 mL, applied over the duration of the root canal preparation) and ethylenediaminetetraacetic acid (15%; 2 mL, 1 min).
3. Adjuvant disinfection in accordance with one of the following three group-specific protocols:
  (a) Additional rinsing with 5 mL sodium hypochlorite (3%; Speiko–Dr. Speier GmbH, Bielefeld, Germany) for 1 min.
  (b) Laser irradiation (SiroLaser Blue; Dentsply Sirona) in continuous-wave mode, at a power setting of 0.6 W for 4 × 10 s with an attached 200-μm fiber tip (Easy Tip Endo; Dentsply Sirona) (Figure 1).
  (c) A combination of sodium hypochlorite rinsing and laser irradiation using the same settings as described above (a + b).

Final rinsing with sodium hypochlorite was then performed in all groups before a calcium hydroxide paste (Calcicur; Voco GmbH, Cuxhaven, Germany) was applied to the prepared root canal, and the tooth was sealed with a foam pellet and glass ionomer cement (Ketac Cem; 3M Espe, Seefeld, Germany).

If the tooth remained symptom-free for 1 week, the temporary filling was removed, and the root canal was obturated with a gutta-percha filling. Collected samples were transferred to transport vessels and sent to an external laboratory for microbiological analysis. The assignment of patients to the different experimental groups was predetermined by the study plan and could not be influenced by the operator or the patient. A total of 57 patients (*n* = 19 in each group) were included in the study.

### 2.3. Laser Application

To ensure consistent laser application, the laser fiber was bent by 45°. Before laser application in the root canal, the active power output was checked for a device power setting of 0.6 W using a power meter (PM100D; Thorlabs GmbH, Bergkirchen, Germany). The device setting of 0.6 W corresponds to an effective power of 0.59 W. Assuming a Gaussian laser beam profile [14], the power density was 3760 W/cm^2^ (fiber tip diameter 0.2 mm, 0.58 W output power).

### 2.4. Microbiological Analysis

The samples were analyzed in a microbiological laboratory (Oro-Dentale Mikrobiologie ODM, Kiel, Germany) using a quantitative real-time polymerase chain reaction (qPCR) after DNA extraction and quantification of the microbiological samples. The main parameter for the analysis was the total bacterial load (TBL), given in genome equivalent colony-forming units (CFUs) per milliliter in accordance with internal laboratory standards. In addition, the bacteria *Porphyromonas endodontalis*, *Treponema denticola*, *Tannerella forsythia*, *Fusobacterium nucleatum*, *Porphyromonas gingivalis*, *Prevotella intermedia*, *Peptostreptococcus micros*, and *Enterococcus faecalis* were analyzed separately in all samples.

### 2.5. Statistical Analysis

A power analysis was performed prior to the study to estimate the number of subjects required on the basis of a study by Blome et al. [4]. For a Cohen effect size of 0.85 [15] and an alpha error of 0.05, an actual power of 0.8 resulted in a total number of 18 root canals per group. The recorded data were transferred to an Excel spreadsheet (Microsoft Corporation, Redmond, WA, USA), and afterwards, statistical analysis was performed using IBM SPSS Statistics for Windows, version 26.0 (IBM Corporation, Armonk, NY, USA). The normal distribution of the values was assessed using the Shapiro–Wilk test. As not all of the data were normally distributed, values were analyzed using a nonparametric test (Kruskal–Wallis) and with Mann–Whitney pairwise comparisons. Comparisons within each study group were performed using nonparametric tests for related samples (Friedman and Wilcoxon tests). Sequentially rejective Bonferroni correction of the critical *p*-value was used when multiple statistical tests were performed simultaneously on a single dataset. Differences were considered statistically significant at *p* < 0.05. Box plot diagrams show the median, first and third quartiles, and minimum and maximum values (whiskers). Values of more than 1.5–3 times the interquartile range (IQR) are specified as outliers and marked as data points. Values more than three times the interquartile range are specified as distant outliers and marked as asterisks.

## 3. Results

At baseline, there were no statistically significant differences in the numbers of bacteria detectable in the root canals in the different groups (*p* > 0.05). Even after chemomechanical root canal preparation, there were no statistically significant differences between the study groups (*p* > 0.05). After the subsequent disinfection procedure in accordance with the study protocol, a statistically significant reduction in bacteria (*p* < 0.05) was observed in all three groups. Since not all bacterial species were consistently detected at the time points specified in the study protocol, a systematic evaluation of the individual species was not performed. The total bacterial load (TBL) in the investigated root canal was therefore used as the main parameter for the analysis.

**Figure 2:**
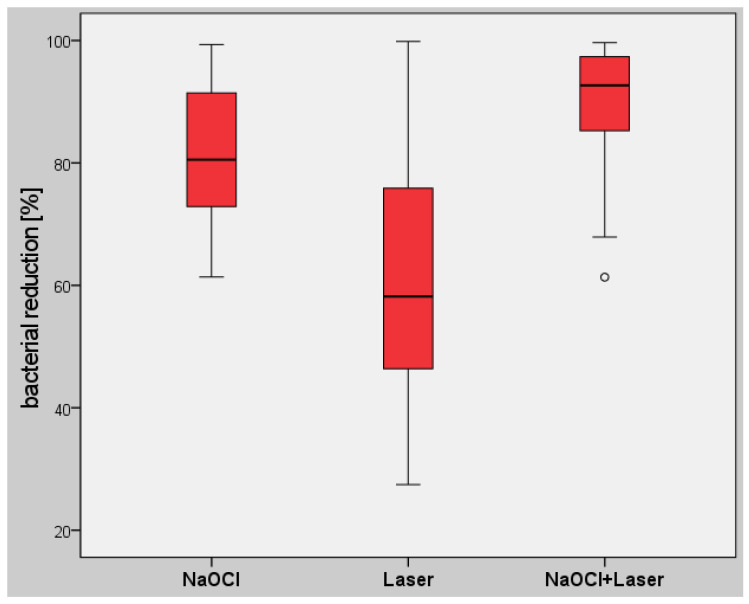
Box plot diagram for the percentage reduction in the total bacterial load (TBL) in the different study groups, showing statistically significant differences between all groups (*p* < 0.05).

In group (a), additional rinsing with sodium hypochlorite resulted in a median TBL of 4.03 × 10^4^ CFU (min. 1.98 × 10^2^, max. 2.36 × 10^6^, IQR 4.59 × 10^4^), representing a percentage bacterial reduction of 80.54% (Table 1 and Table 2). Laser irradiation alone, in group (b), resulted in a median TBL of 5.43 × 10^4^ CFU (min. 5.79 × 10^2^, max. 3.13 × 10^6^, IQR 1.05 × 10^5^). This represented a reduction of 58.18%. In group (c), with a combination of sodium hypochlorite rinsing and laser irradiation, a median total bacterial load of 2.11 × 10^4^ CFU (min. 7.31 × 10^2^, max. 4.18 × 10^5^, IQR 3.52 × 10^4^) was observed, corresponding to a reduction of 92.69% from the baseline value. All of the values for bacterial reduction were thus statistically significant (*p* < 0.05), with the largest reduction in the study group (c) (Figure 2).

## 4. Discussion

The use of rinsing solutions such as sodium hypochlorite (NaOCl) and ethylenediaminetetraacetic acid (EDTA) is regarded as the gold standard for disinfection in endodontic treatments due to their good antibacterial efficacy and ability to remove the smear layer. However, conventional rinsing can be affected by anatomical features and mechanical problems in the conventional rinsing process. The vapor lock effect should be mentioned here, involving the formation of air bubbles in the canal lumen, particularly in the apical area, which impedes the penetration of irrigation solutions [12]. Another major limitation of the disinfection effect with conventional rinsing solutions, which has been widely discussed in the literature, is their limited depth of penetration into the dentin surrounding the root canal. Studies have demonstrated that microorganisms can invade the periluminal dentin up to a depth of 1100 µm [6,8]. However, penetration depths of no more than 160 µm into the dentin have been reported for chemical irrigants used during endodontic treatment procedures [6,7,16]. Such irrigants are therefore unable to eliminate bacteria that have penetrated into the deeper dentin layers [16], and this may lead to recurrent endodontic lesions.

Various adjuvant approaches have been suggested to improve disinfection during root canal treatment procedures. Some of the disadvantages can be partly solved using ultrasound activation or photoactivation systems that enhance penetration and lead at least to some improvement in the antimicrobial activity of rinsing solutions [12]. Laser-based methods have also been developed in recent years and have been reported to be effective for root canal disinfection [16].

The Nd:YAG laser, with a wavelength of 1064 nm, was one of the first lasers used for root canal disinfection [17]. Nowadays, diode lasers with wavelength ranges of around 660–680 nm and 940–980 nm are mainly used for the purpose, and studies have not confirmed adequate bacterial reduction with these [16,17,18,19,20]. Recently, wavelengths in the blue light range have also been investigated in dentistry. High-intensity blue light (405 nm) has been shown to be an effective antimicrobial agent and has displayed significant antimicrobial activity in relation to the ESKAPE bacterial pathogens (*Enterococcus* spp., *Staphylococcus aureus*, *Klebsiella pneumoniae*, *Acinetobacter baumannii*, *Pseudomonas aeruginosa*, and *Enterobacter* spp.), as well as *S. epidermidis*, *S. pyogenes,* and *Candida albicans* [21].

One possible antibacterial effect of laser irradiation is based on the thermal properties of the laser–tissue interaction [16,22]. It is important to keep in mind that the killing of bacteria is not primarily due to the heating of the tooth structure itself. Due to the favorable absorption spectrum of the wavelength of 445 nm, the laser light can penetrate the surrounding root dentin almost unhindered. Due to the high level of absorption into the color components of the bacteria, the laser energy is selectively absorbed and released locally as heat, causing the bacteria themselves to be killed by the increase in temperature. An antibacterial effect can thus also be achieved even in deep tissue layers and at the base of the dentinal tubules [16,23]. In the apical area, where the surrounding dentin is only very thin, laser irradiation is also thought to reach the apical inflammatory processes. Through stimulation of certain enzymes such as alkaline phosphatase, which is involved in bone formation, a positive influence on the regeneration of bony structures has also been reported to be possible [24,25]. In a recently published study, penetration depths of 1000 μm into the depth of the surrounding root canal dentin—comparable to those with Nd:YAG lasers [17,26,27]—have been described with laser light in the blue wavelength range [23]. This is significantly greater than the penetration depth with conventional rinsing solutions.

The limitations of conventional rinsing solutions mentioned above thus do not apply to disinfection with laser light, since penetration into the dentin by laser light makes it possible to eliminate microorganisms in areas of the canal that cannot be reached with conventional rinsing solutions, such as lateral or secondary canals, and in the depths of the dentinal tubules [16,17,18,23].

To achieve the greatest possible effect, the laser light has to be brought as close as possible to the desired target tissue. The diameter of the optical fiber systems is therefore important. Fibers with a diameter of around 200 µm, as used in the present study, allow the laser light to be applied in the depths of the root canal, which is an important prerequisite for the penetration of the laser light into the surrounding dentin [16,17]. The helical movement from apical to coronal carried out in the present study allows extensive irradiation of the canal walls but may still have limitations in the form of irradiation gaps. To eliminate this disadvantage, fiber delivery systems that allow lateral light emission and thus circumferential irradiation of the canal wall should be developed in order to further improve the antibacterial effects [28,29].

In the present study design, the multistage root canal treatment with a bacteria-proof temporary filling corresponds to the standard treatment used to treat possible acute exacerbations after root canal preparation as quickly as possible [30]. In accordance with the experimental design, conventional sodium hypochlorite and EDTA rinsing were used in defined quantities during chemomechanical treatment in all of the groups so that the influence of the additional disinfection measures being investigated was the only variable among the different study groups. The results of the present study support previous findings suggesting that laser irradiation should be regarded as an adjuvant disinfection protocol. Studies have shown that the combination of conventional irrigation fluids and laser irradiation can achieve more extensive disinfection than conventional irrigation fluids alone [17,19]. An in vitro study by Katalinić et al. showed that the laser protocols tested, including 445-nm laser irradiation, were not able on their own to completely eradicate the microorganisms investigated. Instead, they were recommended for clinical usage as an adjunct to conventional NaOCl rinsing [31].

It should be mentioned that bacterial reduction in the present study was investigated only in the root canal itself. It can be assumed that, in addition, differences in the effectiveness of the various disinfection methods are likely to be more significant in deeper layers of the dentin. Due to the greater penetration of laser light into root canal dentin, which has been demonstrated in previous studies [18,23], bacterial reduction in the deeper dentin layers can be expected with the combination of laser light and NaOCl, in addition to the reduction of bacteria in the canal lumen itself. As the eradication of persistent microorganisms even in distant areas of the tubular system is a major challenge in today’s endodontic treatment regimens, this aspect should be carefully evaluated in future studies.

In view of the impossibility of completely sterilizing the endodontic system, there is a consensus that reducing the residual bacteria in the root canal below a pathologically relevant level that can be controlled by the immune system is essential for positive treatment response, allowing root canal treatment to be clinically and radiologically successful [32]. However, it should always be kept in mind that residual bacteria can lead to a recurrence of previously treated endodontic infections if the activity of the immune system declines due to age or disease, and that long-term treatment success may not be guaranteed if the patient’s general health is impaired. The maximum possible bacterial reduction should, therefore, always be a priority during endodontic therapy. Adjunctive laser applications appear to provide stronger and deeper disinfection of the root canal system.

## 5. Conclusions

The present study indicates that conventional chemomechanical root canal preparation allows extensive bacterial reduction. Laser irradiation alone did not show comparable antibacterial efficacy, but the combination of conventional root canal treatment and adjunctive 445-nm laser irradiation resulted in significantly higher levels of bacterial reduction. It can therefore be assumed that adjuvant disinfection using 445-nm laser light can usefully supplement conventional approaches to systematic root canal treatment. However, the clinical impact of increased bacterial reduction on the long-term success of endodontic treatment needs to be evaluated during a clinical follow-up of the treated cases.

## Figures and Tables

**Table 1:** Total bacterial load in CFU/mL in the study groups at the different time points for sample collection.

	Baseline	Root Canal Preparation	Adjunctive Disinfection
NaOCl (Group a)			
Mean	5.74 × 10^6^	5.14 × 10^5^	1.98 × 10^5^
Standard Deviation	2.32 × 10^7^	1.26 × 10^6^	5.54 × 10^5^
Median	1.84 × 10^5^	9.25 × 10^4^	4.03 × 10^4^
Minimum	3.06 × 10^4^	8.81 × 10^2^	1.98 × 10^2^
Maximum	1.01 × 10^8^	4.48 × 10^6^	2.36 × 10^6^
Interquartile Range	9.75 × 10^4^	1.30 × 10^5^	4.59 × 10^4^
*n*	19	19	19
**Laser Irradiation (Group b)**			
Mean	2.97 × 10^6^	5.12 × 10^5^	2.27 × 10^5^
Standard Deviation	9.75 × 10^6^	1.59 × 10^6^	7.05 × 10^5^
Median	2.03 × 10^5^	9.81 × 10^4^	5.43 × 10^4^
Minimum	1.14 × 10^3^	1.02 × 10^3^	5.79 × 10^2^
Maximum	4.18 × 10^7^	7.00 × 10^6^	3.13 × 10^6^
Interquartile Range	2.36 × 10^5^	1.85 × 10^5^	1.05 × 10^5^
*n*	19	19	19
**NaOCl + Laser Irradiation (Group c)**			
Mean	1.72 × 10^6^	1.89 × 10^5^	5.64 × 10^4^
Standard Deviation	3.94 × 10^6^	5.06 × 10^5^	1.00 × 10^5^
Median	2.00 × 10^5^	4.83 × 10^4^	2.11 × 10^4^
Minimum	1.11 × 10^4^	2.29 × 10^3^	7.31 × 10^2^
Maximum	1.49 × 10^7^	2.26 × 10^6^	4.18 × 10^5^
Interquartile Range	1.50 × 10^5^	9.24 × 10^4^	3.52 × 10^4^
*n*	19	19	19

**Table 2:** Percentage reductions in the total bacterial load in each study group.

	NaOCl (Group a)	Laser (Group b)	NaOCl + Laser Irradiation (Group c)
Mean	80.61	63.06	89.40
Standard Deviation	12.32	21.98	10.43
Median	80.54	58.18	92.69
Minimum	61.38	27.46	61.35
Maximum	99.35	99.87	99.67
Interquartile Range	18.58	29.51	12.08
*n*	19	19	19

The numbers for the percentage reduction follow a common rule of three of the basic data set: [100 − (100/base value) × final value].
